# A Nationwide Study of Severe and Protracted Diarrhoea in Patients with Primary Immunodeficiency Diseases

**DOI:** 10.1038/s41598-017-03967-4

**Published:** 2017-06-16

**Authors:** Wen-I Lee, Chien-Chang Chen, Tang-Her Jaing, Liang-Shiou Ou, Chuen Hsueh, Jing-Long Huang

**Affiliations:** 1 Primary Immunodeficiency Care and Research (PICAR) Institute, Chang Gung Memorial Hospital, Chang Gung University College of Medicine, Taoyuan, Taiwan; 2 Division of Allergy, Asthma, and Rheumatology, Department of Pediatrics, Chang Gung Memorial Hospital, Chang Gung University College of Medicine, Taoyuan, Taiwan; 3 Division of Gastroenterology, Department of Pediatrics, Chang Gung Children’s Hospital, Taoyuan, Taiwan; 4 Division of Hematology/Oncology, Department of Pediatrics, Chang Gung Memorial Hospital, Taoyuan, Taiwan; 5 Department of Pathology, Chang Gung Memorial Hospital, Taoyuan, Taiwan

## Abstract

Diarrhoea lasting longer than 14 days and failing to respond to conventional management is defined as severe and protracted diarrhoea (SD). In this study, we investigated the prevalence, pathogens and prognosis of SD in primary immunodeficiency diseases (PIDs). Among 246 patients with predominantly paediatric-onset PIDs from 2003–2015, 21 [Btk (six), IL2RG (four), WASP, CD40L, gp91 (three each), gp47, RAG2 (one each)] and five [CVID (four), SCID (one)] without identified mutations had SD before prophylactic treatment. Detectable pathogens included pseudomonas, salmonella (six each), *E. coli*, cytomegalovirus, coxsackie virus and cryptosporidium (one each), all of whom improved after a mean 17 days of antibiotics and/or IVIG treatment. Seven (7/26; 27.0%) patients died [respiratory failure (four), lymphoma, sepsis and intracranial haemorrhage (one each)]. The patients with WAS, CGD and CD40L and SD had a higher mortality rate than those without. Another five males with mutant XIAP, STAT1, FOXP3 (one each) and STAT3 (two) had undetectable-pathogenic refractory diarrhoea (RD) that persisted >21 days despite aggressive antibiotic/steroid treatment and directly resulted in mortality. For the patients with RD without anti-inflammatory optimization, those with mutant XIAP and FOXP3 died of Crohn’s-like colitis and electrolyte exhaustion in awaiting transplantation, while transplantation cured the STAT1 patient.

## Introduction

The intestinal tract is the largest lymphoid organ in the body and contains lymphocytes, macrophages, and dendritic cells to prevent pathogenic invasion, modulate inflammation^1, 2^, and produce sufficient antibodies for optimal neutralization, opsonization, and complement activation^3, 4^. If these cellular and humoral immune responses do not effectively cooperate and mutually balance, unwanted or excess reactions will injure the epithelium, mucosa, submucosa, and connective tissue, consequently leading to persistent diarrhoea despite nothing per os, hydration, antibiotics and immunosuppressant therapy^1–3^.

Severe and protracted diarrhoea (SD) is generally defined as prolonged diarrhoea lasting for more than 2 weeks, usually emerging within 2 years of life and requiring parenteral nutrition^5^, although the definition has undergone several revisions to better delineate the duration and clinical course^6^. Some studies have emphasised that specific aetiologies including infections that persist for longer than expected should be included in the definition.

Excluding congenital enterocyte defects^7, 8^, SD is often accompanied by “little effect of treatment”, “failure to gain weight”, and/or co-morbidity with “recurrent infections”, all of which are warning signs of primary immunodeficiency diseases (PIDs)^9^. Increasing evidence suggests that SD can mimic inflammatory bowel disease (IBD) and that it is associated with PIDs patients who have defective IL-12/23^10–16^ and IL-10 signalling^17–21^, profound T cell defects^22–29^, nicotinamide adenine dinucleotide phosphate oxidase anomalies^30, 31^, anti-apoptosis signalling of X-linked inhibitor of apoptosis (XIAP)^32–35^, and nuclear factor kappa B transcription (NEMO) signalling^36^. Research linking SD to monogenetic PIDs has shed light on the pathogenesis, and suggests that hematopoietic stem cell transplantation (HSCT) can be a potential cure, especially if there is profound T and polymorphonuclear cell deficiencies leading to recurrent opportunistic life-threatening infections and serious persistent intestinal inflammation.

With increased susceptibility to infections and inflammation, PIDs patients can develop the SD phenotype and present with frequent diarrhoea for longer than 14 days despite conventional management^1, 3^. In this nationwide study from the referral institute of Primary Immunodeficiency Care and Research (PICAR), we investigated the prevalence, pathogens and prognosis of PIDs patients with the SD phenotype.

## Results

### Enrolled Patients

Of the enrolled patients, 26 (2 females) had the SD phenotype, including the Btk gene in six, IL2RG in four, WASP in three, CD40L in three, gp91 in three, gp47 in one, RAG2 in one, and four with CVID and one with SCID without identified genetic defects. The mutations included splicing, deletion, missense and stop mutations, but not insertion mutations (Table 1). Their median age at onset of the SD phenotype was 14 months (range 2–273 months; mean ± S.D. 48.6 ± 65.0 months), with a median duration of diarrhoea of 14 days (range 14–18 days; mean 15.0 ± 1.6 days), median length of total parental nutrition (TPN) of 7 days (range 4–10 days; mean 7.0 ± 2.0 days), and median length of antibiotic treatment of 16.5 days (range 16–20 days; mean 16.9 ± 1.7 days) including ceftriaxone, a combination of ceftazidime and amikacin, and carbapenems and/or meropenem. Neutropenia was noted in four patients (Btk1-2, Btk3, Btk5 and SCID2-IL2RG) with pseudomonas colitis and two (SCID3-IL2RG and CGD4-gp47) with salmonella colitis. These patients also developed bronchiectasis (11), sepsis (7), and significant other events (21) before adequate prophylactic treatment which included sulfamethoxazole/trimethoprim and fluconazole for T cell deficiency, sulfamethoxazole/trimethoprim, itraconazole, and/or interferon-gamma for polymorphonuclear cell deficiency, regular immunoglobulin infusion for B cell deficiency, and ampicillin-sulbactam for bronchiectasis.

**Table 1:** Severe and protracted diarrhoea in the patients with PIDs who responded to antibiotics.

Gender	Tested age (months)	Genetic mutations	Colitis pathogen	Effective treatment [IgG level mg/dL at episode] (diarrhoea/treatment duration, days)**	TPN	Associated symptoms		
					(days)	Bronchiectasis	Sepsis	Other significant events (mortality, cause)
**Predominantly antibody deficiencies**								
Btk1-1/M	40	Btk: Int 14 (−2)A > G; skip exon 14	undefined	IVIG [93], CTZ, AMK (15/16)	7	+	+	
Btk1-2/M	14	Btk: Int 14 (−2)A > G; skip exon 14	Pseudomonas	IVIG [102], CTZ, AMK (14/15)	7			
Btk2/M	9	Btk: c.1821C > T; p. Arg 641 Cys	Pseudomonas	IVIG [92], CTZ, AMK (14/15)	8		+	Recurrent cellulitis
Btk3/M	36	Btk: c. 1042 T > G; p. Phe304Val	Pseudomonas	IVIG [107], CTZ, AMK (14/15)	8			Recurrent sinopulmonary infections
Btk4/M	110	Btk: c.232 C > T; p. Glu78Stop	Salmonella	IVIG [205], CTX, CAR (14/16)	7			
Btk5/M	26	Btk: c.1562 A > T; p. Asp 521 Val	Pseudomonas	IVIG [78], CTZ, AMK (17/19)	9		+	Polyarthritis, facial cellulitis
CVID1/M	273	Undefined*	undefined	IVIG [263], CTX, AMK (18/20)	10	+		Recurrent sinopulmonary infections
CVID2/M	127	Undefined*	undefined	IVIG [169], CTZ, AMK (15/17)	5	+		Recurrent sinopulmonary infections, failure to thrive
CVID3/M	129	Undefined*	undefined	IVIG [205], CTZ, AMK (14/16)	4	+		Recurrent sinopulmonary infections, Takayashi arteritis
CVID-4/F	92	Undefined*	Salmonella	IVIG [187], CTX (15/18)	5			Recurrent sinusitis, otitis media
**Combined immunodeficiencies**								
SCID1-IL2RG/M	10	IL2RG: c. 220 T > G; p. Trp74Gly	*E. coli*	IVIG [104], CTX (14/17)	8		+	PJP, BCG-related infection, stem cell transplantation
SCID2-IL2RG/M	6	IL2RG: c. 676 G > T; p. Arg226Cys	Pseudomonas	IVIG [99], CTZ, AMK (14/16)	6			BCG-related infection, stem cell transplantation
SCID3-IL2RG/M	3	IL2RG: c. 865 C > T; Arg289Stop	Salmonella	IVIG, CTX (14/15)	5			Interstitial pneumonitis (respiratory failure)
SCID4-IL2RG/M	3	IL2RG: c.854 G > A, skip exon 6	Cytomegalovirus	IVIG [107], CTX, Gancyclovir (20/21)	12		+	Pneumonitis (respiratory failure)
SCID5/M	2	undefined	undefined	IVIG [245], CTZ, AMK (15/17)	7	+		Pneumonitis, pulmonary haemorrhage, (respiratory failure)
SCID6-RAG2/F	4	RAG1: Leu474Arg, Arg776Gln	undefined	IVIG [542], CTX, AMK (14/16)	7			Stem cell transplantation
HIGM1-CD40L/M	121	CD40L: Del 347 A	Cryptosporidium	IVIG [213], CTX, AMK (15/18), nitazoxanide (15/18)^	10	+		PJP, Recurrent sinopulmonary infections
HIGM2-CD40L/M	3	CD40L: c.526 T > A; p.Tyr 169 Asn	Salmonella	IVIG [87], CTX (17/19)	8	+		
HIGM3-CD40L/M	5	CD40L: c.526 T > A; p.Tyr 169 Asn	*E. coli* Coxsackievirus B4	IVIG [112], CTX, AMK (14/15)	4	+	+	Cholecystitis, cholangitis, mortality (lymphoma)
**Combined immunodeficiencies with associated or syndromic features**								
WAS1/M	3	WASP: Del promoter, exon 1 and 2	undefined	IVIG [421], CTX, AMK (14/17)	7		+	Bloody diarrhoea, mortality (Staphy. aureus sepsis)
WAS2/M	3	WASP: Del 243–250 nu	undefined	IVIG [354], CTX, AMK (14/16)	5			Bloody diarrhoea, severe atopic dermatitis, mortality (intracranial haemorrhage)
WAS3/M	2	WASP: c. 91 G > A, p. Glu31Lys	undefined	IVIG [743], CTX, AMK, MEP (18/20)	10			Bloody diarrhoea, stem cell transplantation
**Congenital phagocyte number, function or both**								
CGD1-gp91/M	31	Gp91: Del 1693G	IBD-like	CTX, AMK, prednisolone (14/17)	5	+		Aspergillosis, pneumatocele, BCG-related infection
CGD2-gp91/M	14	Gp91: c.1028 C > A, p.Thr343Lys	Salmonella	CTX (14/15)	5			Staphy. aureus lymphadenitis, perianal ulcers, stem cell transplantation
CGD3-gp91/M	94	Gp91: c.1249 G > T, p. Gly412Val	Salmonella	CTX (14/16)	7	+		Aspergillosis, BCG-related infection, (respiratory failure)
CGD4-gp47/M	102	Gp47: del 75, 76GT (exon 2)	Pseudomonas	CTZ, AMK (14/17)	7	+		Aspergillosis, recurrent oral ulcers

Abbreviations: CAR: carbapenems (100 mg/kg/day, divided q 6 hours); CTX: ceftriaxone (100 mg/kg/day); CTZ: ceftazidime (100 mg/kg/day); AMK: amikacin (15 mg/kg/day); IVIG: intravenous immunoglobulin (0.5–0.8 g/kg/dose); MEP: meropenem (20–30 mg/kg/day, divided q 12 hours); PJP, Pneumocystis jirovecii pneumonia; BCG, Bacillus Calmette-Guerin. “+” means “with” bronchiectasis or/and sepsis

*The eight genes of inducible costimulator (ICOS), transmembrane activator and calcium modulator and cyclophilin ligand interactor (TACI), CD19, B-cell activating factor receptor (BAFFR), CD81, CD20, CD21, Cytotoxic T-lymphocyte–Associated antigen 4 (CTLA-4) and ﻿LPS-responsive beige-like anchor (LRBA) were analysed, but all were wild type.

**ceftriaxone (100 mg/kg/day) was given for suspected Salmonella or Shigella colitis, and a combination of ceftazidime (100 mg/kg/day) and amikacin (15 mg/kg/day) for Pseudomonas colitis before the pathogen cultures were available.

^Nitazoxanide (200 mg/day bid) was discontinued when frequent diarrhoea had subsided and the stool culture became negative.

**Figure 1:**
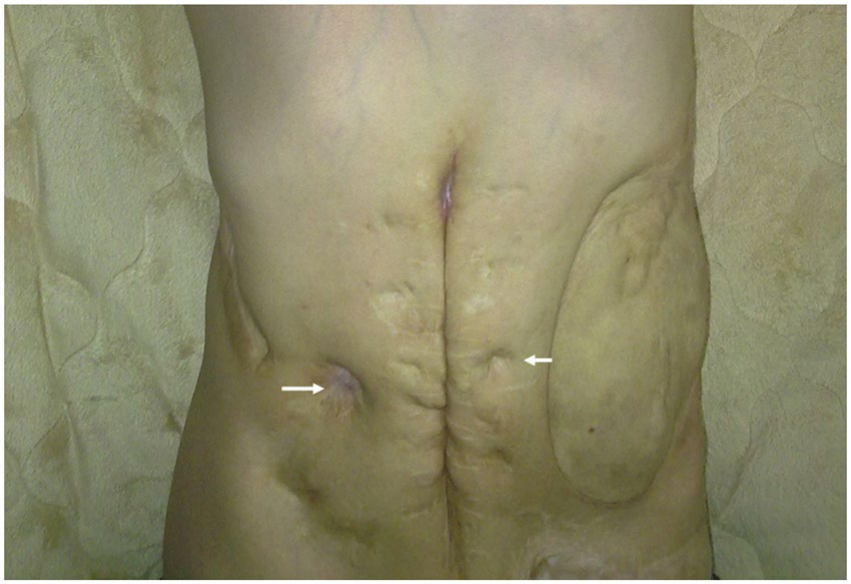
The morbidity of refractory severe and protracted diarrhoea (RD) in patient 4 was caused by a cutaneous peritoneal intestinal fistula.

**Figure 2:**
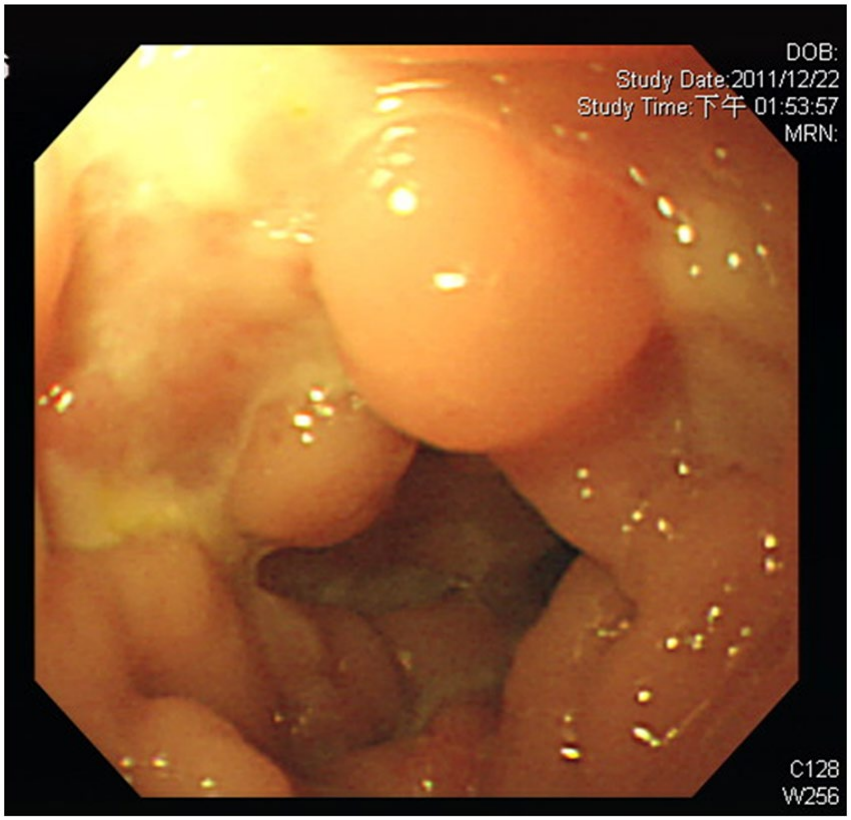
Colonofiberoscopy revealed a cobblestone mucosal pattern and multiple pseudo-polyp-like lesions.

**Figure 3:**
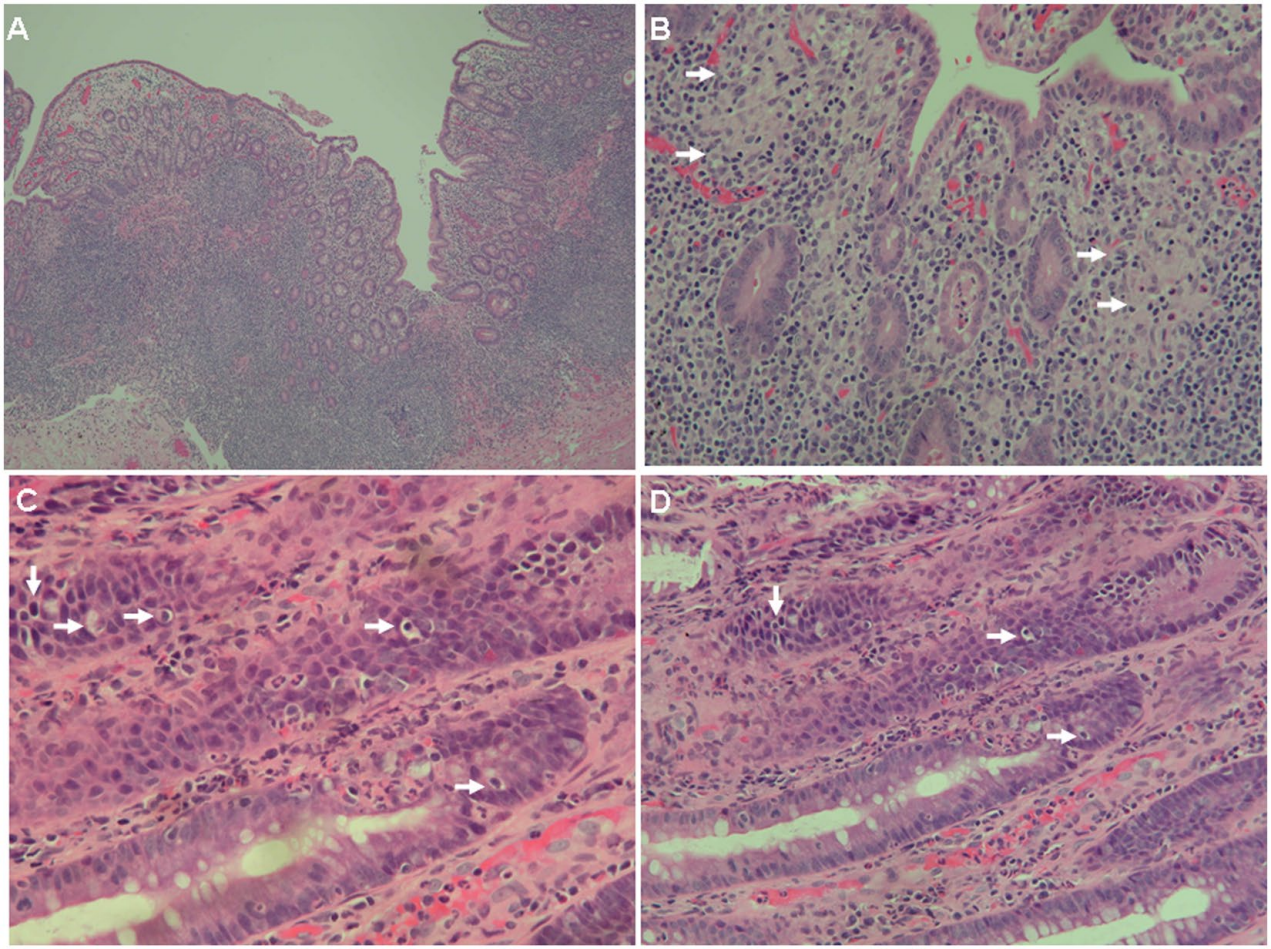
(**A**) Small intestine biopsy of patient 1 revealed marked villous atrophy and acute and chronic inflammation (hematoxylin-eosin [H&E] staining, magnification 100X) and (**B**) granulomatous inflammation (white arrows) composed of aggregates of epithelioid histiocytes in the lamina propria (H&E, 200X). (**C**,**D**) Colon biopsy of patient 1 showed ulceration and acute and chronic inflammation, with prominent epithelial apoptosis (white arrows) and intra-epithelial neutrophils (H&E, 400X).

**Figure 4:**
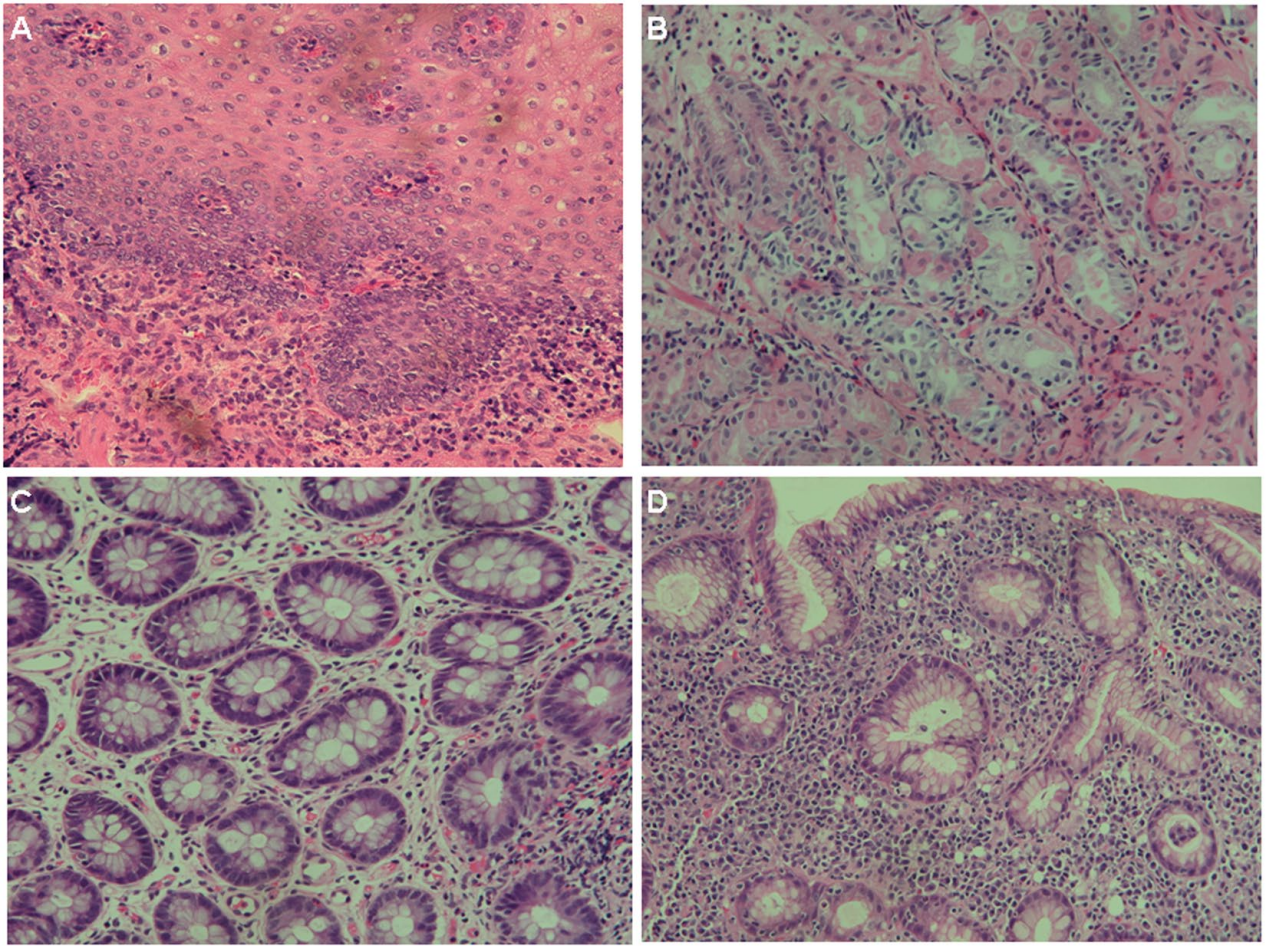
An endoscopic biopsy in patient 2 demonstrated (**A**) moderate lymphocytic infiltrates in the oesophagus (H&E staining, 200X) and **(B)** paucity of plasma cells in the stomach (H&E, 400X). (**C**) In patient 3, repeated colonic biopsies disclosed mildly increased lymphocytes and decreased plasma cells without granuloma, ulcer, or cryptitis (H&E, 400X). (**D**) A gastric biopsy in patient 5 revealed chronic active gastritis and colonization of Helicobacter pylori (H&E, 200X).

Another five male patients had a poor response to intravenous immunoglobulin (IVIG) and empiric antibiotics plus steroids over 3 weeks, and were defined as having the refractory diarrhoea (RD) phenotype (Table 2) with blood-stained diarrhoea but without obvious protein-losing enteropathy. Four patients had a peri-anal abscess without fistula formation, and one (patient 4) had a perforated colon and severe pyoderma in the abdominal wall that damaged his rectus muscularis. Peritoneal reconstruction with a 10 × 10 cm skin graft was performed to cover the injured abdominal wall, however he was complicated by an intestinal peritoneal cutaneous fistula (Fig. 1). Anaemia, elevated ESR, and hypoalbuminemia indicated chronic inflammation, consistent with the coloscopic observation (Fig. 2) and the pathological findings (Figs 3 and 4).

**Table 2:** Gastrointestinal and associated clinical features of the patients with PIDs and refractory diarrhoea.

Genetic mutation	Patient 1 XIAP Int5(+1)G > A	Patient 2 STAT1 T385M	Patient 3 FOXP3 M370L	Patient 4 STAT3 Int10(−2)A > G	Patient 5 STAT3 G469R
**Gastrointestinal ﻿manifestations﻿﻿ (“+” means﻿ positive findings; “-” means negative findings)**					
Gender/Onset/Present age	M/10 M/2Y8M (died)	M/1 M/13Y2M	M/NB/4 M (died)	M/5Y/14Y6M	M/15Y2M/19Y3M
Frequency at worst per day	16	11	12	7	8
Bloody-stain	+	+	+	+	+
Protein-losing enteropathy	−	−	−	−	−
Failure to thrive	+	+	+	−	−
Peri-anal abscess	+	+	+	−	+
Fistula	−	−	−	+	−
Endoscopic pathology*					
Oesophagus	No definite lesion	Isolated tiny erosions	No definite lesion	No definite lesion	Erythematous mucosa
Stomach and duodenum	No definite lesion	Scattered erosions at antrum	No definite lesion	No definite lesion	Erythematous mucosa with some erosions at stomach. Ulcer with granulation at duodenal bulb
Jejunum and ileum	Multiple segments of wall thickening and skipped lesion	No definite lesion	No definite lesion	Perforation and inflammation	Mild inflammatory process at proximal jejunum
Colon	Cobblestone mucosal pattern and multiple pseudo-polyp-like lesions with aphthous ulceration. Much whitish to yellowish exudates coating on oedematous mucosa.	Some oedematous mucosa	Scattered hyperaemic and oedematous mucosa	Chronic inflammation and perforation	Scattered hyperaemic and oedematous mucosa
**Medication^ for diarrhoea**					
	Empiric antibiotics, IVIG, prophylactics, methylprednisolone, TPN (10 M)	Empiric antibiotics, IVIG, prophylactics, methylprednisolone, stem cell transplantation, TPN (2Y11M)	Empiric antibiotics, IVIG, prophylactics, methylprednisolone, sirolimus, TPN (3 M)	Empiric antibiotics and IVIG, if flare-up, methylprednisolone, TPN (2Y8M)	Empiric antibiotics and IVIG if flare-up, methylprednisolone, TPN (2 M)
**Associated symptoms**					
(Onset age)	Incomplete HLH (4 M)	MRSA pustulosis (1 M)	Jaundice caused by TPN-relayed	Severe pyoderma and colon perforation	Recurrent MRSA cellulites and carbuncles (2Y)
	Norovirus enteritis (10 M)	BCG-induced lymphadenitis (7 M)	cholestasis (1 M)		
	Refractory anaemia and thrombocytopenia (1Y)	Refractory pneumonia (7 M) Bronchiectasis (10 M)			
	Splenectomy (1Y)	Intermittent oral candidiasis			
	Sepsis by Klebsiella pneumonia, extended spectrum β lactamase (ESBL) *E. coli*, and Candida (1Y) Pneumo-peritoneum from the perforated colon (1Y)	Hepato-splenomegaly (4 M)			
**Significant laboratory findings**					
Hb mg/dL (>10)	10.2	**9.8**	**9.6**	11.1	13.2
ESR /min (<45)	**45.0**	**56.1**	**47.2**	**58.2**	5.0
Albumin mg/dL (>3.5)	**2.2**	3.5	**1.7**	3.8	4.1
Liver function					
AST (13–40 U/L)	**340**	**63**	**53**	**44**	13
ALT (<36 U/L)	**257**	27	**45**	25	21
**Immune assessments**					
Neutrophil (2100–4520/mm3)	**1040**	4361	4456	4095	4460
Absolute lymphocytes (2000–6500 /mm3)	2600	**245**	3176	3549	1934
CD4 (31–56%)	34.6	34.2	44.5	31.9	38.7
CD4CD45RA (12–45%)	19.3	**9.1**	31.4	20.1	25.7
CD8 (12–35%)	37.2	**1.5**	35.4	24.3	33.2
CD4 memory (**)	15.2	24.5	**12.2**	**11.4**	17.2
CD19 (6–27%)	20.3	31.4	15.2	26.6	12.8
CD19 memory (**)	**1.0**	**0.7**	2.5	**0.3**	**1.2**
NK (3–22%)	6.1	5.3	3.1	5.8	9.7
IgM (49–156 mg/dL)	358	252	63	219	92
IgG (334–1230 mg/dL)	527	1072	673	1130	877
IgA (15–113 mg/dL)	40	**434**	39	63	136
IgE (<100 IU/ml)	**254**	16	**2920**	**45900**	**4940**
Lymphocyte proliferation (cpm)					
PHA 2.5 ug/ml (29228–58457)	**284**	**1453**	45721	32156	24783
PWM 0.1 ug/ml (11395–28487)	**280**	**787**	21529	15786	22146
Candida 2.5 ug/ml (5351–13328)	**41**	**53**	10142	8514	9327
BCG 0.002 ug/ml (1740–4352)	**18**	**16**	2143	4023	3417
Superoxide production (86–99%)	87.4	94.5	94.6	89.7	91.3
TNF-α suppression (4.7–21.4%)	7.5	5.0	18.1	10.9	8.4

Abbreviations: cpm, counts per minute; M in sex, male; Y and M in tested age, years and months; MRSA, Methicillin-resistant Staphylococcus aureus .

*Oesophageal, gastric, and duodenal lesions were demonstrated by endoscopy; small intestinal lesions were revealed by small bowel series with contrast medium; colonic lesions were demonstrated by colonofiberoscopy.

^Empiric antibiotics refer to ceftriaxone (100 mg/kg/day) for presumed Salmonella or Shigella colitis; ceftazidime (100 mg/kg/day) and amikacin (15 mg/kg/day) for presumed Pseudomonas colitis although not proven in stools and blood cultures. The prophylactics given to patient 1, 2, and 3 were fluconazole (5 mg/kg/day) as anti-fungal treatment and co-trimoxazole (trimethoprim 5 mg/kg/day) as anti-pneumocystis jirovecii pneumonia treatment. Immunosuppressants of prednisolone (1–2 mg/kg/day) and sirolimus (3 mg/m2) were given.

**Note: The percentage of memory CD4 + cells was calculated from CD4 + multiple [CD4 + CD45RO + /CD4 + CD45RA + plus CD4 + CD45RO + ], while the percentage of memory CD19 + cells was from CD19 + multiple [CD19 + CD27 + /CD19 + CD27 + plus CD19 + CD27−].

Normal ranges of the percentage of memory CD4+ cells were 3–26% in infants between 3 months and 3 years of age (from 10 healthy infants) and 18–57% in children over 3 years of age (from 8 healthy children). Normal ranges of the percentage of memory CD19+ cells were 1.4–2.4% in infants between 3 months and 3 years of age (from 11 healthy infants) and 1.4–6.6% in children over 3 years of age (from 9 healthy children). Underlined bold numbers represent values below the normal ranges, while bold numbers represent values above the normal range.

### Immunologic Studies and Genetic Analysis

The characteristics of PIDs patients with the SD phenotype (Table 1) including micro-thrombocytopenia, recurrent sinopulmonary infections, hypogammaglobulinemia, catalase-positive pathogens, opportunistic infections, and lymphopenia were recognized as Wiskott-Aldrich syndrome (WAS), X-linked agammaglobulinemia (XLA), X-linked hyper IgM syndrome (HIGM), common variable immunodeficiency (CVID), chronic granulomatous disease (CGD), severe combined T- and B-cell immunodeficiency (SCID), according to the genetic mutations of the updated PIDs classification^37, 38^.

The immunologic evaluation of these five patients with RD (Table 2) showed that one had neutropenia, one had decreased absolute lymphocytes, naïve CD4, and CD8 cells, two had decreased memory CD4 cells, and four had decreased memory CD19 B cells. Quantitative levels of IgE were increased in four patients and the level of IgA was increased in one. Lymphocyte proliferation was decreased in two patients, while four had impaired liver function. Superoxide production from stimulated PMNs and suppression of TNF-α production through IL-10 signalling in LPS-treated PBMCs were within normal range.

Of note, patient 1 also had extreme splenomegaly and recurrent hemophagocytic lymphohistiocytosis with a high EBV viral load^32–35^. He had a splicing mutation at intron 5 (+1) G > A (Supplementary Fig. 1A), missing exon 5, frameshift at the 354^th^ Val ending at the 379^th^ due to the lack of the RING (Really Interesting New Gene) domain in the XIAP gene (Supplementary Fig. 1B) to inhibit caspase 3 or 7 activity. His mother was a carrier. Patient 2 had a STAT1 mutation impairing the IFN-γ-IL-12/23 circuit, thereby increasing susceptibility to mycobacterial and Candida infections. A [Thr385Met] STAT1 mutation in the DNA-binding domain region also caused an IPEX-like phenotype (immune dysregulation, polyendocrinopathy, enteropathy, and X-linked)^39–41^. This genetic mutation was finally identified 8 years post-transplantation (Supplementary Fig. 1C). Patient 3 had the IPEX phenotype, which had caused the death of his maternal uncle due to diabetes. His [Met370Leu] FOXP3 mutation changed the fork-head domain and decreased the percentage of Treg cells (CD25 + + CD4+/CD4+, 5.8% vs. 32.6%) (Supplementary Fig. 1D and E). In patients 4 and 5, extremely high IgE levels and recurrent cutaneous infections were the distinct manifestations of hyper IgE syndrome. The STAT3 mutations were found to have IVS 10 (−2) A > G leading to a splicing loss of exon 11 and missense mutation of Gln469Arg in exon 15 (Supplementary Fig. 1F and G, respectively, in the DNA-binding domain region). Both were de novo loss-of-function mutations and their family members did not have the hyper IgE syndrome phenotype.

### Treatment Response and Prognosis

Salmonella and pseudomonas strains were the most common of 17 identified pathogens in the patients with the SD phenotype (in 6 and 6 patients, respectively). Even though cytomegalovirus and cryptosporidium were only isolated in one patient with SCID4-IL2RG and one with HIGM1-CD40L, both had persistent cramping pain and high CRP levels, implying possibly mixed bacterial pathogens in such opportunistic infections. Both patients improved with effective antibiotic treatment plus gancyclovir and nitazoxanide, respectively. Giardia and clostridium difficile toxin A were not detected in any of our patients. In addition to IVIG infusion for those with hypogammaglobulinemia according to age, those with the SD phenotype improved within 3 weeks by effective and empritic antibiotics. However, even though all of the patients with RD without hypogammaglobulinemia received consistent treatment including IVIG and empiric antibiotics plus methylprednisolone over 3 weeks, only a limited effect was achieved. The exception was patient 4 who had a STAT3 mutation, in whom the number of RD flare-ups decreased from 5–6 episodes per year to 1–2 episodes per year after receiving steroids and regular IVIG treatment. During his episodes, the frequency was an average 5–6 times per day lasting for 15–22 days.

Seven of the 26 patients (27.0%) with SD died of respiratory failure (four), and lymphoma, sepsis, and intracranial haemorrhage (one each) rather than directly of severe diarrhoea. Compared to the patients with the same PIDs without the SD phenotype (Table 3 and Supplemental Fig. 2), those with XLA and SCID with the SD phenotype trended to have a lower mortality rate. In contrast, the patients with WAS, CGD and HIGM with the SD phenotype had a relatively higher mortality rate, although none of the differences reached statistical significance except XLA patients.

**Table 3:** The relationship between mortality and the SD phenotype (severe and protracted diarrhoea**)** in each group of patients or whether directly related to SD or RD (refractory diarrhoea) phenotype.

Diseases	With SD phenotype		Without SD phenotype		Statistic test *p* value
	Alive	Death	Alive	Death	Kalpan-Meier survival*
	(patient number)		(patient number)		
XLA	6	0	4	2	**0.0486**
SCID	3	3	7	9	0.5122
WAS	1	2	15	4	0.066
CGD	3	1	15	2	0.5623
HIGM	2	1	10	0	0.3173
Total	15	7	51	17	0.9230
**Mortality**	**SD phenotype**		**RD phenotype**		
	**Non-related**	**Related**	**Non-related**	**Related**	
	7	0	0	2	

*Kalpan-Meier survival analysis defined the diagnosis day as the first follow-up day to compare those PIDs patients with and without the SD phenotype. For those without SD, the starting time of K-M analysis was the diagnosis day by characteristic presentations or/and molecular and genetic confirmation. They had no the SD phenotype to date. For those with SD, the starting time of K-M analysis was the diagnosis day by characteristic presentations accompanying SD or/and molecular and genetic confirmation.

In contrast to the SD group, the higher mortality rate in the RD group (two deaths of five patients; 40%) was directly related to their overwhelming RD phenotype. The parents of patient 1 (with an XIAP mutation) chose a conservative strategy rather than HSCT despite a poor response to aggressive treatment and methylprednisolone. Anti-TNF-α biologics were considered, however the RD phenotype worsened and endemic norovirus enterocolitis prevailed. Unfortunately, he died of severe Crohn’s colitis and accelerated hemophagocytic lymphohistiocytosis. Patient 3 (with a FOXP3 mutation) succumbed from recurrent RD while waiting for a suitable HSCT donor. Patient 2 (with a STAT1 mutation) underwent successful HSCT to terminate recurrent opportunistic and life-threatening infections as well as the RD phenotype. Patient 5 had broader gastrointestinal tract lesions from the stomach to the colon and lost 3 kg after having diarrhoea for 2 months. Concomitant Helicobacter pylori colonization was found in the second endoscopic stomach specimen, and additional amoxicillin and clarithromycin (2 weeks) and omeprazole (12 weeks) treatment attenuated the cramping and frequent diarrhoea.

## Discussion

This is the first study to report the prevalence, distribution, pathogens and prognosis of SD from a national PIDs referral centre. Twenty-six patients (10.6%) who had insufficient opsonized and neutralized IgG (Btk, CVID, SCID and HIGM), diminished reactive oxygen species production (CGD), and lazy lymphocytes with cytoskeletal dysfunction (WAS), thus impairing pathogenic eradication developed the SD phenotype before adequate prophylactic therapy and needed an extended duration of antibiotic treatment. Severe bacterial colitis in immune-competent patients often responds to effective antibiotics within 7 days in Taiwan in the previous study^42^. Of note, pseudomonas colitis was most commonly found in the patients with predominantly antibody deficiencies, of whom one (Btk2) met the diagnostic criteria^43^ of Shanghai fever including community-acquired diarrhoea with fever, sepsis and pseudomonas cultures from facial ecthyma gangrenosum. His pathogen was not a multi-drug resistant strain.

In the pathogenesis of the extreme presentation of the RD phenotype, persistent intestinal inflammation seemed to play a greater role than infection, reflecting the poor response to antibiotics. Therefore, alternative biologics or anti-inflammatory immuno-suppressants stronger than steroids should be administered to patients with XIAP, STAT1, FOXP3, or STAT3 mutations. While alimentary enterocytes with these mutations interact with microorganisms for gut homeostasis, the XIAP mutation selectively impairs nucleotide-binding and oligomerization domain 1/2 signalling for chemo-attractant production (IL-6, IL-8, and MCP-1), and does not effectively terminate cognate caspase-related cascades for apoptosis in damaged and/or infected intestinal epithelium^32–35^. The dominant gain-of-function hypermorphic [Thr385Met] STAT1 mutation prolongs STAT1 phosphorylation to exaggerate IFN-γ, IFN-α, IL-6, and IL-21 cytokine signaling^39–41^. The FOXP3 mutation in Treg cells attenuates IL-10 and TGF-β production to release unwanted autoantibodies in autoimmune disorders^17–21^. In addition, we previously showed that the STAT3 mutation diminishes Th17 production^44^, inhibiting the intestinal tight junction^45, 46^, epithelial cell proliferation, goblet cell restoration, and mucin production^47, 48^. Furthermore, a novel suppressive Th17 subset has been shown to cause regulatory Th17 (rTh17) cells to induce mucosal tolerance when Th17 cells are recruited to the intestinal tract^49^. Taken together, the pathologic mechanisms orchestrated by these mutations break intestinal mucosal tolerance and augment persistent intestinal inflammation, thereby causing the RD phenotype. Successful HSCT is, in practice, rescue therapy to terminate the RD phenotype in patients with XIAP, FOXP3 and STAT1 mutations as well as recurrent infections^50^.

Helicobacter pylori-infected gastric mucosa can express higher levels of IL-17 via the STAT3 pathway^51^ and promote intestinal metaplasia and dysplasia to carcinoma in PIDs patients in whom malignant transformation has been reported to be 10 times higher than the general population^52^. However, the lack of IL-17 secretion in patients with the loss-of-function STAT3 mutation due to defective Th17 cell development does not enhance alimentary malignant transformation to induce the tumour-secreting-endocrine RD phenotype. Whether the Helicobacter pylori infection in our patient 5 with a STAT3 mutation was related to the RD phenotype is unclear, and further large-scale prospective studies are warranted to elucidate this issue.

There are several limitations to this study. First, some aetiological pathogens and triggering factors of SD were not identified, even though comprehensive PCR and stool cultures were performed to detect viruses, fungi, parasites and bacteria. Clearly delineating between infection and inflammation remains a challenge in PIDs patients with immune disturbances. Hence, both empiric antibiotics and steroids should be recommended to those with the RD phenotype. Second, in three RD patients with FOXP3, XIAP, and STAT1 mutations, successful HSCT reconstructed immunity and therefore resolved the RD phenotype as well as the recurrent infections. However, only one patient with the STAT1 mutation received timely HSCT. The hesitation of the other two parents to undergo HSCT increased the mortality rate despite an early diagnosis and aggressive treatment. Third, the prevalence of IBD in PIDs patients is under-estimated. IBD or IBD-like diarrhoea may emerge at an older age since the life expectancy of PIDs patients has increased due to advanced treatment strategies, thereby increasing the potential to develop inflammation and autoimmune disorders. The limited follow-up duration of our patients with predominantly paediatric-onset PIDs may explain the lower prevalence of IBD or IBD-like diarrhoea.

In conclusion, the 26 (10.5%) patients in this nationwide cohort of 246 PIDs patients were mostly male and developed the SD phenotype before adequate prophylactic treatment. Most cases were caused by salmonella and pseudomonas infections, and they needed IVIG and an extended period of antibiotic treatment. Five (2.0%) male patients with the RD phenotype had XIAP mutations resulting in defective nucleotide-binding and oligomerization domain 1/2 signalling, the hypermorphic STAT1 mutation causing hyper-cytokine signalling, and profound Treg and Th17 cell defects caused by FOXP3 and STAT3 mutations. Early recognition and timely effective interventions to suppress inflammation are beneficial to decrease the mortality rate of PIDs patients with the RD phenotype.

## Methods

### Patients

From 2003 to 2015, 246 index cases with PIDs from 215 families were identified from the PICAR Institute’s registry (Supplemental Table 1)^37^. Infectious colitis was diagnosed if the clinical manifestations of bloody diarrhoea, tenesmus, severe cramping abdominal pain and a high CRP level (>40 mg/mL) were unequivocal even if no pathogen was isolated after extensive culture and PCR amplification. According to our previous studies on the epidemiologic intestinal pathogens in patients with PIDs^37, 53^, ceftriaxone (100 mg/kg/day) or meropenem (30 mg/kg/day) was given for suspected Salmonella or Shigella colitis, and a combination of ceftazidime (100 mg/kg/day) and amikacin (15 mg/kg/day) or carbapenem (100 mg/kg/day) for pseudomonas colitis in the presence of neutropenia resembling Shanghai fever was given within the first 24 hours before the pathogen cultures were available^43^. When the blood cultures were sterile and infectious manifestations of tenesmus, severe cramping abdominal pain, fever, and elevated CRP level subsided, the antibiotics were discontinued.

Regardless of whether empiric or sensitive antibiotics were given for the identified pathogens and IVIG (0.6–0.8 g/kg) for the patients with hypogammaglobulinemia, frequent diarrhoea of more than three episodes of unformed stools within a period of 24 hours for longer than 2 weeks was defined as the SD phenotype. In cases of frequent diarrhoea that failed to respond to antibiotics, one dose of IVIG and steroids (methylprednisolone 2–5 mg/kg/day) for more than 3 weeks was defined as the refractory SD (RD) phenotype in this study. The prognosis of the patients with PIDs suffering from SD and RD were compared using Kalpan-Meier survival (GraphPad software) tests. For those without SD, the starting time of K-M analysis was the diagnosis day by characteristic presentations or/and molecular and genetic confirmation. They had no the SD phenotype to date. For those with SD, the starting time of K-M analysis was the diagnosis day by characteristic presentations accompanying the SD phenotype or/and molecular and genetic confirmation.

The Chang Gung Human Investigation Committee approved all carried methods and all experimental protocols in this study, and the patients’ parents or guardians’ informed consent were obtained. We confirmed that all methods were performed in accordance with the relevant guidelines and regulations.

### Immunologic Functional Assessment and Candidate Gene Approach

In addition to immunoglobulin levels and lymphocyte subpopulations (i.e., CD3+, CD4+, CD4 + CD45RA+, CD4 + CD45RO+, CD19+, CD19 + CD27+, and CD19 + CD27−), characteristics and well-syndromes of PIDs accompanying the SD phenotype were identified by experienced physicians, and were consistent with defective lymphocyte proliferation to mitogens and antigens, and phorbol myristate acetate-stimulated polymorphonuclear reactive oxygen species as represented by H_2_O_2_ production as previously described^54^.

To evaluate IL-10 signalling in the patients with RD and unrecognized molecular defects ﻿at﻿ t﻿hat time﻿, purified PBMCs (5 × 10^6^/ml) were stimulated overnight with 50 ng/ml *E. coli* LPS (Sigma-Aldrich, St. Louis, Mo) alone or with 20 ng/ml recombinant human IL-10 (R&D Systems, Minneapolis, MN). The supernatants were analysed using a commercially available TNF-α ELISA development kit in duplicate using a Tecan Sunrise ELISA micro-plate reader (R&D Systems, Minneapolis, MN). Candidate genetic analysis in the patients with the RD phenotype predisposing to IBD included, at least, IL10, IL10RA, IL10RB, NEMO, FOXP3, XIAP, STAT3 and STAT1^55, 56^. Every two oligonucleotide primers were selected to cover the entire coding region by Sanger sequencing^57^.

## Electronic supplementary material

Supplemental information
