# Supplementary material for: A Nationwide Study of Severe and Protracted Diarrhoea in Patients with Primary Immunodeficiency Diseases
Source: Sci Rep. 2017 Jun 16;7:3669. doi: 10.1038/s41598-017-03967-4 (PMC5473906; doi:10.1038/s41598-017-03967-4)

**A Nationwide Study of Severe and Protracted Diarrhoea in Patients with Primary Immunodeficiency Diseases**

Wen-I Lee, MD, PhD^a,b*^; Chien-Chang Chen, MD^c*^; Tang-Her Jaing, MD^b,d^; Liang-Shiou Ou, MD^b^; Chuen Hsueh MD^e^; and Jing-Long Huang, MD^a,b*^;

**Supplemental Table 1.** Distribution patterns in patients with PIDs (n=246; 215 unrelated families)

|  | | | **Reported** | | | | **Died** | | |  | |
| --- | --- | --- | --- | --- | --- | --- | --- | --- | --- | --- | --- |
| **Disease category** |  | | **F** |  | **M** |  | | **F** | **M** |  |  |
| **Predominate antibodies deficiencies** |  |  | **19** |  | **38** |  | | **0** | **2** |  |  |
| Common variable immunodeficiency (CVID) |  |  | 12 |  | 16 |  | |  |  |  |  |
| Agammaglobulinemia |  |  | 1 |  | 2 |  | |  |  |  |  |
| X-linked agammaglobulinemia (XLA) |  |  | 0 |  | 12 |  | |  | 2 |  |  |
| Selective immunoglobulin deficiency ^a^ |  |  | 6 |  | 3 |  | |  |  |  |  |
| Transient hypogammaglobulinemia of infant (THI) |  |  | 0 |  | 5 |  | |  |  |  |  |
| **Combined immunodeficiencies** |  |  | **9** |  | **31** |  | | **4** | **10** |  |  |
| Severe combined B and T-cell immunodeficiency (SCID) |  |  | 7 |  | 15 |  | | 4 | 8 |  |  |
| Omenn syndrome |  |  | 1 |  | 1 |  | |  |  |  |  |
| Hyper IgM syndrome (HIGM) |  |  | 0 |  | 13 |  | |  | 1 |  |  |
| Combined immunodeficiencies with predominant T-cell defect ^b^ |  |  | 1 |  | 1 |  | |  | 1 |  |  |
| (MHC Class I deficiency) ^c^ |  |  | 0 |  | 1 |  | |  |  |  |  |
| **Congenital defects of phagocyte number, function or both** |  |  | **8** |  | **27** |  | | **1** | **5** |  |  |
| Chronic granulomatous disease (CGD) |  |  | 2 |  | 19 |  | |  | 3 |  |  |
| Chronic severe neutropenia |  |  | 4 |  | 2 |  | |  |  |  |  |
| Interferon-γ associated immunodeficiency ^d^ |  |  | 1 |  | 3 |  | |  |  |  |  |
| Leukocyte adhesion disease (LAD) |  |  | 1 |  | 1 |  | | 1 | 1 |  |  |
| Bater syndrome |  |  | 0 |  | 1 |  | |  |  |  |  |
| Natural killer cell deficiency ^f^ |  |  | 0 |  | 1 |  | |  | 1 |  |  |
| **Combined immunodeficiencies with associated or syndromic features** |  |  | **28** |  | **61** |  | | **3** | **11** |  |  |
| DiGeorge syndrome (DGS) |  |  | 16 |  | 25 |  | | 1 | 2 |  |  |
| Wiskott-Aldrich syndrome (WAS) |  |  | 1 ^e^ |  | 21 |  | |  | 6 |  |  |
| Hyper IgE syndrome (HIES) |  |  | 7 |  | 4 |  | | 1 | 2 |  |  |
| Ataxia telangiectasia (DNA breakage associated syndrome; AT) |  |  | 2 |  | 4 |  | | 1 | 1 |  |  |
| Comel-Netherton syndrome |  |  | 2 |  | 4 |  | |  |  |  |  |
| Cartilage-hair hypoplasia |  |  | 0 |  | 1 |  | |  |  |  |  |
| Chronic mucocutaneous candidiasis |  |  | 0 |  | 1 |  | |  |  |  |  |
| Primary CD4 T cell deficiency ^f^ |  |  | 0 |  | 1 |  | |  |  |  |  |
| **Disease of immune dysregulation** |  |  | **2** |  | **4** |  | | **2** | **2** |  |  |
| Chediak-Higashi syndrome (CHS) |  |  | 1 |  | 1 |  | | 1 |  |  |  |
| Familial hereditary hemophagocytosis |  |  | 1 |  | 0 |  | | 1 |  |  |  |
| Lymphoproliferative syndrome X-linked |  |  | 0 |  | 1 |  | |  |  |  |  |
| Immunodeficiency polyendocrinopathy enteropathy X-linked |  |  | 0 |  | 2 |  | |  | 2 |  |  |
| **Defects in innate immunity** |  |  | **0** |  | **2** |  | |  | **2** |  |  |
| Anhidrotic ectodermal dysplasia with immunodeficiency (EDA-ID) |  |  | **0** |  | **2** |  | |  | **2** |  |  |
| **Auto-inflammatory disorders** |  |  | **1** |  | **1** |  | |  |  |  |  |
| Cold-induced auto-inflammatory disease (CIAS) |  |  | 1 |  | 1 |  | |  |  |  |  |
| **Complement deficiencies** |  |  | **6** |  | **9** |  | |  | **1** |  |  |
| Primary C3 deficiency |  |  | 0 |  | 1 |  | |  |  |  |  |
| Primary C7 deficiency |  |  | 1 |  | 1 |  | |  |  |  |  |
| Hereditary angioedema |  |  | 5 |  | 7 |  | |  |  |  |  |
| **Total** |  |  | **73** |  | **173** |  | | **10** | **33** |  |  |

Abbreviations: PIDs, primary immunodeficiency diseases; F, female; M, male

^a^Selective immunoglobulin deficiency included IgA (1 female), IgG2 sub-class (2 females, 1 male), IgG2 and IgG4 sub-class (1 female, 1 male), IgG3 sub-class (1 male), and IgG3 and IgG4 sub-class (2 females) deficiencies. No patients had selective deficiency to polysaccharide.

^b^Commonly referred to as Nezelof syndrome.

^c^The patient had decreased expression of MHC class I compared to normal controls, but normal MHC class II. His family refused further genetic analysis.

^d^Interferon-γ associated immunodeficiency includes patients with mutations of IL-12RB1, IFNGR1, IFNGR2, IL-12p40, STAT1, or NEMO deficiency.

^e^The female Wiskott-Aldrich syndrome variant has previously been reported (Lin and Hsu, 1984).

^f^The two diagnoses of natural killer cell deficiency and primary CD4 T cell deficiency are beyond the nine updated categories (Al-Herz W, et al, 2014) [41]. By lymphocyte subset and clinical presentation, we classified natural killer cell deficiency into phagocyte defects and primary CD4 T cell deficiency with normal immunoglobulin levels and without recurrent sinopulmonary infections into “combined immunodeficiencies with associated or syndromic features”.

**Supplemental Figure 1.** Intron 5 (+1) G>A in the XIAP gene in **(A)** patient 1 led to a splicing mutation for the loss of exon 5, frameshift at the 354^th^ Val stopping at the 379^th^, leading to **(B)** loss of the RING domain. **(C)** Nucleotide 1154 C>T in exon 15 of the STAT1 gene in patient 2 caused a de novo missense mutation of the amino acid Thr substation by Met in the 385^th^ location (T385M). The nucleotide 1110 G>A in exon 10 of the FOXP3 gene in patient 3 caused **(D)** a missense mutation of amino acid Met substation by Leu in the 370^th^ location (M370L), and **(E)** decreased the percentage of Treg (CD4+CD25++) cells. His mother was a carrier. In the STAT3 gene, intron 10 (-2) A>G led to a splicing mutation to lose exon 11, thus **(F)** in-frame of the 10 intervention amino acids in patient 4, and **(G)** de novo 1406th nucleotide of A replaced by G in exon 15 caused a missense mutation of Gln469Arg (Q469R) in patient 5.

**Supplemental Figure 2.** Kalpan-Meier survival curves showed comparison between XLA, SCID, WAS, CGD, HIGM and all of these five PIDs “with” and “without” the SD phenotype.


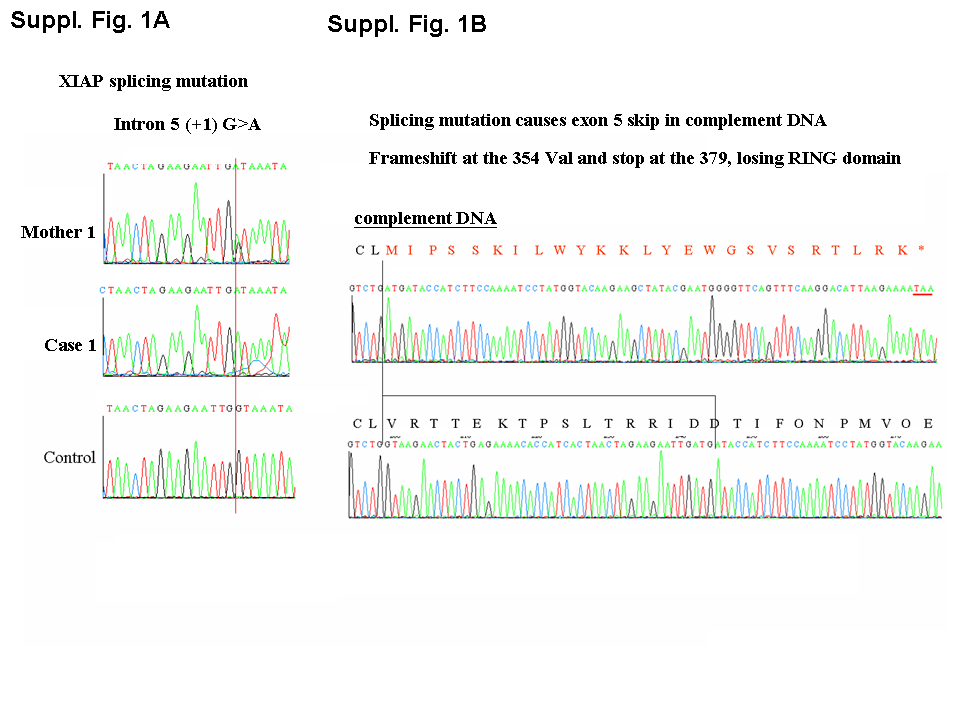


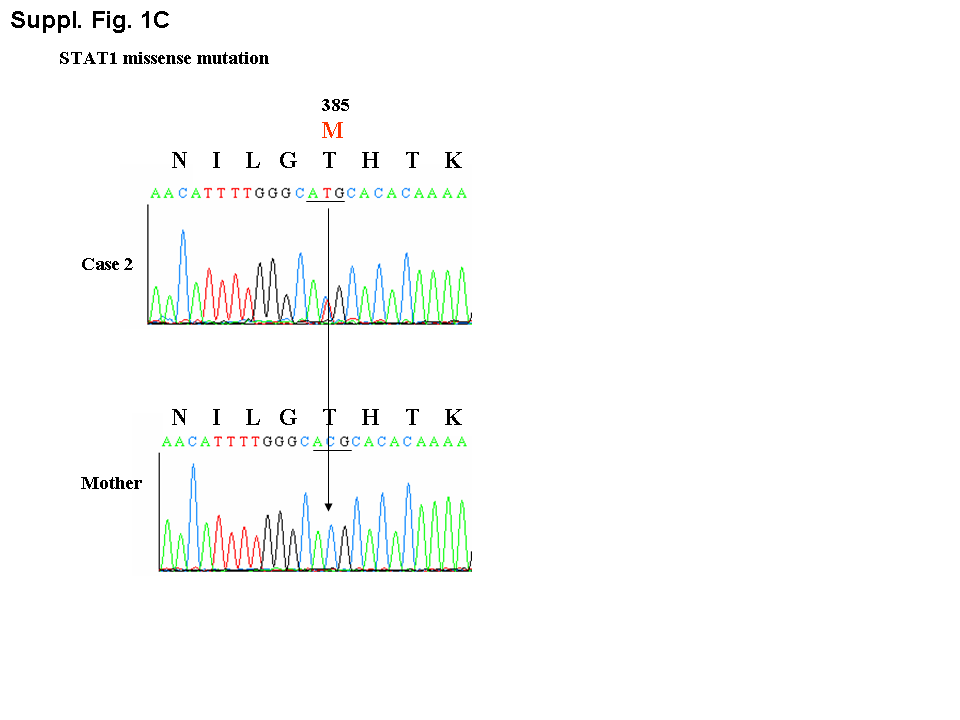


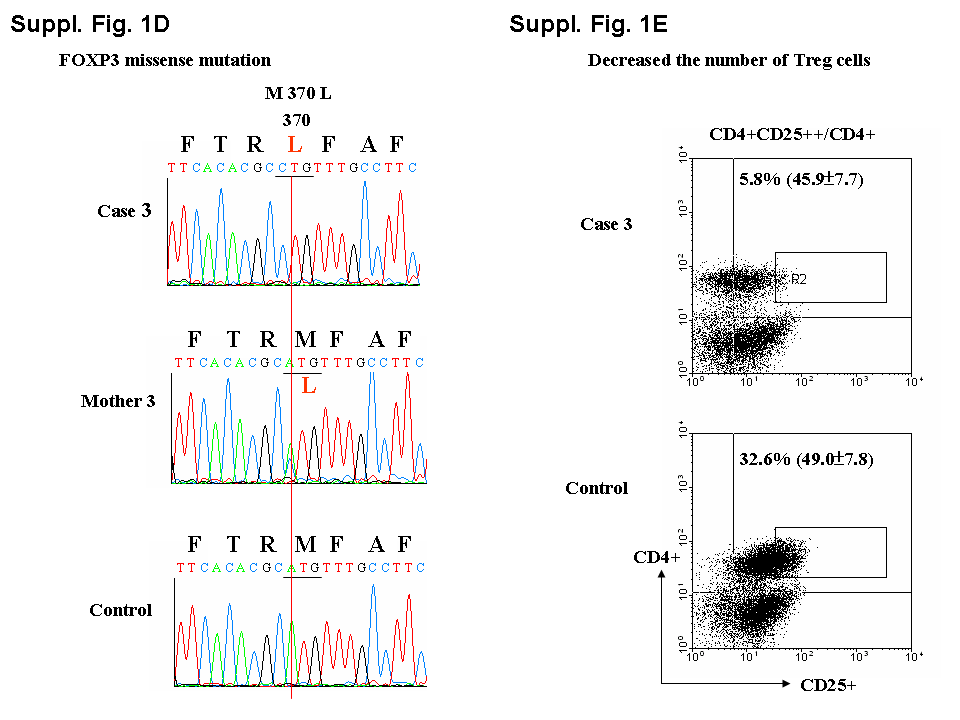


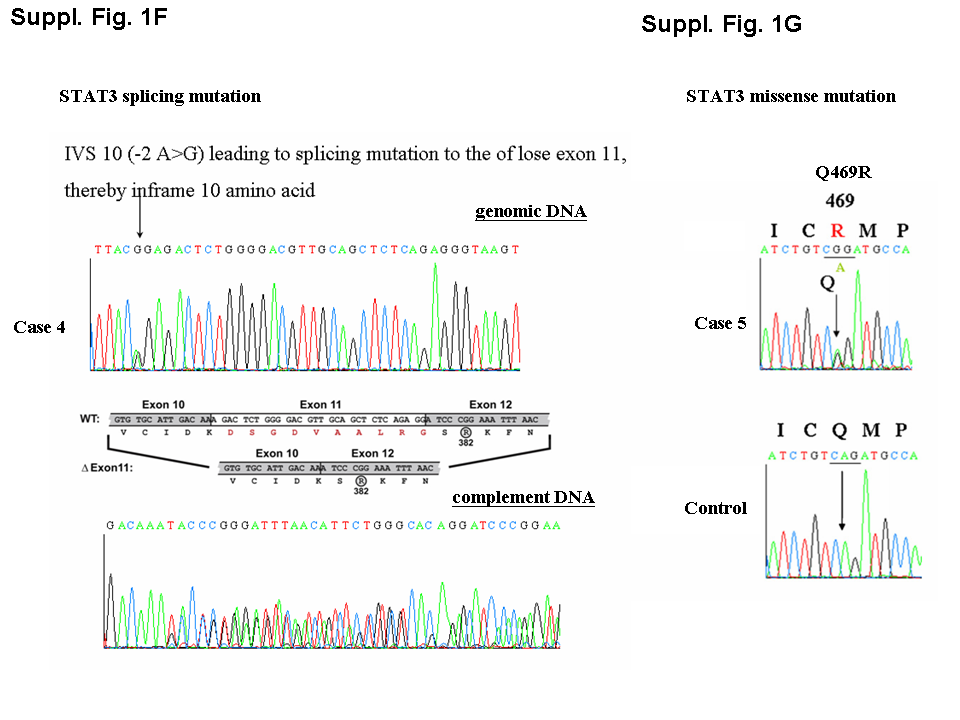


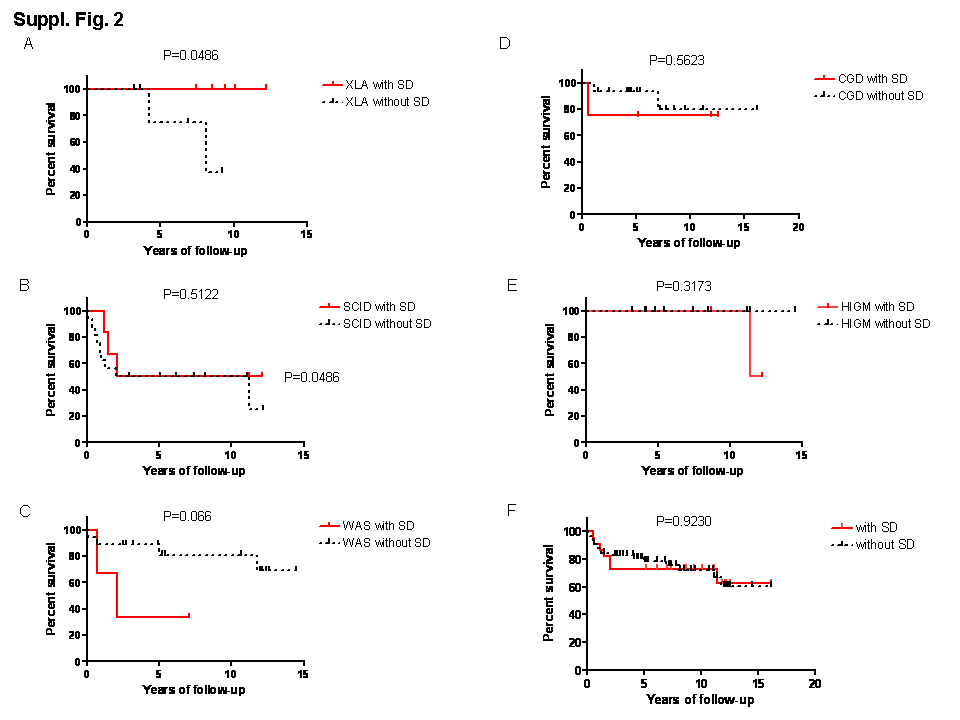

Supplement: Supplementary file 1 — Supplemental information [file 41598_2017_3967_MOESM1_ESM.doc]
